# COVID-19 vaccine acceptance among Bangladeshi adults: Understanding predictors of vaccine intention to inform vaccine policy

**DOI:** 10.1371/journal.pone.0261929

**Published:** 2022-01-13

**Authors:** Clarice Lee, Taylor A. Holroyd, Rachel Gur-Arie, Molly Sauer, Eleonor Zavala, Alicia M. Paul, Dominick Shattuck, Ruth A. Karron, Rupali J. Limaye

**Affiliations:** 1 Department of International Health, Johns Hopkins Bloomberg School of Public Health, Baltimore, MD, United States of America; 2 International Vaccine Access Center, Johns Hopkins Bloomberg School of Public Health, Baltimore, MD, United States of America; 3 Berman Institute of Bioethics, Johns Hopkins University, Baltimore, Maryland, United States of America; 4 Oxford-Johns Hopkins Global Infectious Disease Ethics Collaborative, Oxford, United Kingdom, Baltimore, Maryland, United States of America; 5 Department of Health, Behavior & Society, Johns Hopkins Bloomberg School of Public Health, Baltimore, United States of America; 6 JHU-CCP, Johns Hopkins University Center for Communication Programs, Baltimore; 7 Center for Immunization Research, Department of International Health, Johns Hopkins Bloomberg School of Public Health, Baltimore; 8 Department of Epidemiology, Johns Hopkins Bloomberg School of Public Health, Baltimore, United States of America; The Chinese University of Hong Kong, HONG KONG

## Abstract

**Objectives:**

The purpose of this study was to identify predictors of COVID-19 vaccine intention among Bangladeshi adults.

**Methods:**

Secondary data from the COVID-19 Beliefs, Behaviors & Norms Survey conducted by the Massachusetts Institute of Technology (MIT) and Facebook were analyzed. Data were collected from 2,669 adult Facebook users in Bangladesh and was collected between February 15 and February 28, 2021. Binomial logistic regression examined the relationship between COVID-19 vaccination intent and demographic variables, risk perception, preventive behaviors, COVID-19 knowledge, and likelihood of future actions.

**Results:**

Seventy-nine percent of respondents reported intent to get the COVID-19 vaccine when it becomes available. Intent to get vaccinated was highest among females, adults aged 71–80, individuals with college or graduate-level degrees, city dwellers, and individuals who perceived that they were in excellent health. Results of the binomial logistic regression indicated that predictors of vaccination intent include age (*OR = 1*.*39*), high risk perception of COVID-19 (*OR = 1*.*47*), and intent to practice social distancing (*OR = 1*.*22*).

**Discussion:**

Findings suggest that age, perceived COVID-19 risk, and non-pharmaceutical COVID-19 interventions may predict COVID-19 vaccination intent among Bangladeshi adults. Findings can be used to create targeted messaging to increase demand for and uptake of COVID-19 vaccines in Bangladesh.

## Introduction

On March 11, 2020, the World Health Organization (WHO) declared Coronavirus disease 19 (COVID-19), a new strain of coronavirus, a pandemic [1]. Globally, there have been more than 150 million cases, resulting in more than 3 million deaths [2]. As the result of the development of COVID-19 vaccines at record speed, more than 1.19 billion COVID-19 vaccine doses (16 doses per 100 people) have already been administered worldwide, with the number increasing daily [3].

COVID-19 cases in Bangladesh were first confirmed on March 8, 2020 by the National Institute of Epidemiology, Disease Control and Research [4]. Within Bangladesh, there have been more than 766,000 reported cases, resulting in 11,705 deaths [2]. The country experienced an initial peak of nearly 4,000 cases per day in early July 2020, followed by a mostly declining trend through February 2021. This overall decline in cases in the early months of 2021 is most likely due to public health measures such as a nationwide lockdown, travel bans, and school closures, despite the high population density in Bangladesh [5]. However, Bangladesh started to experience a rapid escalation in cases beginning in March 2021, reaching over 7,000 cases per day in early April 2021, likely driven in part by the emergence and spread of variants of concern in the region [6]. In particular, the Delta variant (B.1.617.2) was first detected in Bangladesh in early May 2021. This highly transmissible variant emerged in late 2020 in India and contributed heavily to that country’s catastrophic surge in cases and deaths in 2021, and has since been detected in countries around the world [7]. Recent analyses by Bangladesh’s Institute of Epidemiology, Disease Control and Research (IEDCR) and partners detected the Delta variant in 80% of 50 samples sequenced in mid-May 2021 [7, 8]. Similar to other countries, risk factors for COVID-19 related mortality in Bangladesh include older age (65+), male sex, and pre-existing morbidities [9, 10].

On February 7, 2021, Bangladesh began administering the Oxford AstraZeneca vaccine AZD1222 to priority groups, including those over 40 years of age and frontline health workers [11]. As of October 2021 end, 25.1% of the population have received a first dose of the vaccine [12]. Bangladesh has also approved two other vaccine products for emergency use: Gam-COVID-Vac (Sputnik V) and BBIBP-CorV (Sinopharm COVID-19 vaccine) [13, 14]. The Bangladeshi government also began inoculating the population with the Sinopharm vaccine in June [15]. The majority of Bangladesh’s vaccine supply to date has been donated by the Government of India [16], with additional shipments from COVID-19 Vaccines Global Access (COVAX) to be expected in June 2021 [17]. COVAX, co-led by WHO, Gavi, and Coalition for Epidemic Preparedness Innovations (CEPI), aims to provide fair and equitable access to COVID-19 vaccines for every country [18]. All participating countries in COVAX have equal access to vaccines supported by COVAX [19]. As vaccine supply increases in Bangladesh, efforts to vaccinate a greater share of the population will need to consider vaccination intent and hesitancy in other population groups.

Few studies have been conducted in Bangladesh assessing intent to vaccinate against COVID-19 [20–23]. According to recent cross-sectional surveys, approximately 69–75% of adults in Bangladesh report at least probable intent to vaccinate against COVID-19; however, willingness to vaccinate varies by several demographic and psychosocial factors [20, 22]. In their study of 3,646 adults, Abedin et al. [20] found vaccine refusal and hesitancy more prevalent among those who live in rural areas and urban slums than those who reside in other urban areas. Results suggest that lower levels of risk-perception may be driving hesitancy, and although findings were not statistically significant, the authors note considerable effects by age group, family income, and educational attainment. Older adults (60+) were more than twice as likely to be unwilling to vaccinate against COVID-19 compared to younger ages, and those with higher income and education are more accepting toward the vaccine [20].

In two pre-print articles, Ali and Hossain [21] and Mahmud et al. [23] report on their investigations related to determinants of vaccine hesitancy among adults in Bangladesh. In Ali and Hossain’s study of 1,134 adults [21], 32% expressed vaccine hesitancy, defined as “delay in acceptance or refusal of vaccination despite the availability of the vaccination service” [24]. Unemployed persons, males, and adults more than 60 years old were more likely to express vaccine hesitancy, which is consistent with Abedin’s findings [20]. In Mahmud et al.’s study [23], 62% preferred to delay COVID-19 vaccination until they either had more information about the vaccine’s safety and effectiveness, or COVID-19 mortality in Bangladesh rose. In contrast, Kabir et al. [22] found no association between COVID-19 vaccination intent and sociodemographic factors. The authors also found that religion played a significant factor in vaccine hesitancy with Muslims being 64% less likely to express definite or probable vaccine intent compared to non-Muslims [22]. They conclude that given the Islamic point of view where preservation of religion comes before preservation of life, vaccine hesitancy may be greater among those concerned that the vaccine is not halal [22].

It is evident that challenges to achieving high rates of vaccine uptake remain. There is a limited supply of vaccine doses, especially in low- and middle-income countries. Furthermore, there is widespread mistrust in vaccine safety and effectiveness globally [25]. Public knowledge and positive attitudes toward vaccines have been consistently associated with vaccine uptake [25]. Nevertheless, research has shown that knowledge does not always correlate with positive vaccine acceptance and behavior [26]. Vaccine uptake is ultimately also impacted by many factors, including personal beliefs, religious or moral values, vaccination access, anecdotes, and previous vaccination experiences [26]. Therefore, it is critical to understand the different factors that could reduce COVID-19 vaccine hesitancy. There is currently limited information assessing intent to vaccinate against COVID-19 within the context of Bangladesh. This information is critical to inform vaccine acceptance strategies and policies. As a result, we sought to identify predictors of COVID-19 vaccine acceptance among Bangladeshi adults to better inform ways in which public health agencies in Bangladesh can encourage vaccine uptake.

## Methods

### Survey design

Data were drawn from the COVID-19 Beliefs, Behaviors & Norms Survey conducted by the Massachusetts Institute of Technology (MIT) and Facebook. This global survey was conducted in 60+ countries to better understand people’s knowledge, beliefs, behaviors, and risk perceptions regarding COVID-19 to help improve policy and communication responses [27]. The survey was first administered on July 7, 2020 and ended on March 28, 2021, with over 2,000,000 responses across all countries.

In Bangladesh, a multi-wave survey ran continuously for multiple two-week waves. During each wave, Facebook aimed to survey 3,000 respondents. Facebook users in Bangladesh (and other countries) were shown a survey advertisement on Facebook. Individuals who clicked on the advertisement were pushed to an independent survey portal hosted by MIT, which asked them to verify their age (over 18 years old) and administered informed consent. No personal information was shared between Facebook and MIT. Once enrolled, all participants were administered the same five blocks of questions on information exposure, knowledge, vaccine and healthcare, and demographics. A randomization scheme limited survey fatigue and randomly assigned other sections of the survey. Thus, no participants answered all the questions in the survey. There was no financial incentive for participating in the survey.

The original study design utilized non-response modeling and poststratification techniques to design sampling and weight methods [27, 28]. A regression and poststratification model was developed to generate survey weights for respondents in each survey wave, based on the inverse probability of responding to the survey [29] providing the most parsimonious survey non-response model to ensure a representative sample of the Facebook user base. These methods helped minimize sampling variability and nonresponse biases. After weighting for non-response, weights were adjusted based on census data to better represent the target population for each country.

For our analysis, we focused on respondents from Bangladesh who took the survey wave between February 15 and February 28, 2021. A total of 4,430 participants completed this wave. As the goal of our study was to understand how to increase vaccine acceptance and policy, we were interested in identifying what factors might make those that had not yet been vaccinated get the vaccine. As a result, we excluded participants who reported that they had already been vaccinated for COVID-19. We also excluded participants who completed less than 80% of the survey. Due to insufficient statistical power, we also excluded individuals who had selected “other” in the response to gender (n = 2). The Committee on the Use of Humans as Experimental Subjects review board at MIT approved the original study [30]. This was a secondary data analysis completed on publicly available data, and as such ethics approval was waived.

### Outcome measure

The survey question “If a vaccine for COVID-19 becomes available, would you choose to get vaccinated?” was used as the outcome variable for this study. Four answer choices were given: “I have already been vaccinated”, “Yes”, “No”, or “Don’t Know”. We excluded those who answered “I have already been vaccinated”. The outcome of vaccine intention was dichotomized, with those that answered yes characterized as “intend to get the vaccine” and those who answered either no or don’t know as “do not intend to get the vaccine”.

### Independent variables

Independent variables in this study included demographic characteristics, risk perception, COVID-19 knowledge, preventive measures taken, and future behaviors. Demographic characteristic variables included age (under 20, 20–30, 31–40, 41–50, 51–60, 61–70, 71–80, over 80), gender (female or male), education (less than primary school, primary school, secondary school, college/university, graduate school), area of dwelling (city, town, village or rural), and perception of one’s own health status (poor, fair, good, very good, excellent). The education variable was re-coded into three categories (less than secondary, secondary, tertiary) for analysis.

Participants were asked how dangerous they think COVID-19 is to their community on a five-point scale from “not all dangerous” to “extremely dangerous”. Participant’s COVID-19 knowledge was determined by two questions on existing treatments and positive cases. Participants were asked about current existing treatments, with five answer choices: “There is a drug to treat COVID-19”, “There is a vaccine for COVID-19”, “There is both a drug for treatment and a vaccine for COVID-19”, “There is currently no drug treatment or vaccine for COVID-19”, and “I am unsure which is correct.” They were also asked whether they personally knew someone who had tested positive for COVID-19 (yes or no).

To collect data on preventive measures, participants were asked what measures they had taken to prevent COVID-19 infection in the past week. Response options included washing hands regularly using disinfectants or soap and water, wearing a face mask or face covering, social distancing, and none. Future public health behaviors were assessed with questions about how likely participants were to wear a mask in public and social distance over the next two weeks, with response options on a five-point scale from “never” to “always”.

Collinearity between variables was evaluated through variance inflation factors (VIFs). VIFs were obtained from a dummy multiple linear regression model fitted with all the independent variables. Results from the model show that the independent variables are not correlated with one another using a VIF cutoff value of 10 (VIFs: 1.04–1.63).

### Statistical analysis

Data were analyzed using Stata 16.1 (College Station, TX: StataCorp LLC). The data was not additionally weighted for analysis. Demographic characteristics of the study sample are presented using descriptive statistics. Cross-tabulations between the outcome (vaccination intention) and demographic characteristics were performed to assess frequencies. A binomial logistic regression model was fitted to examine the relationship between all the independent variables and outcome. While no variable selection procedure was used, variables entered into the adjusted model were selected based on relevance to COVID-19 behaviors and vaccine intention. In addition to fitting the fully saturated model, we performed a stepwise regression, successively adding each variable into the model and balancing significant variables ≤0.05 with parsimony. The coefficients from the adjusted logistic regression model were exponentiated and are expressed as odds ratios (OR) with corresponding 95% confidence intervals (CI).

## Results

A total of 2,669 survey responses were included for analysis. Demographic characteristics of the respondents are provided in Table 1. The median age group of respondents was between 20 and 30 years with 46% of respondents falling in this group. The majority (83%) of respondents were male, and 68% of respondents were urban residents. As for educational attainment status, 87% of respondents had attended either a college/university or graduate school. Only 13% of respondents perceived their own health to be less than good, while 46% of respondents perceived their own health to be very good or excellent.

**Table 1:** Baseline characteristics of the study population (n = 2,671).

Sociodemographic variable	n	%
Gender		
Male	2193	82.1%
Female	444	16.6%
Other	2	0.1%
Education		
Less than secondary	2275	85.7%
Secondary	260	9.8%
Tertiary	96	3.6%
Age		
Under 20	280	10.5%
20–30 yr	1221	45.7%
31–40 yr	616	23.1%
41–50 yr	276	10.3%
51–60 yr	145	5.4%
61–70 yr	65	2.4%
71–80 yr	22	0.8%
Over 80	11	0.4%
Area of dwelling		
City	1758	67.8%
Town	381	14.7%
Village or Rural	443	17.1%
Own Health		
Poor	69	2.6%
Fair	265	10.0%
Good	1073	40.6%
Very Good	741	28.0%
Excellent	457	17.3%

Missing data not included in data. Missing data accounted for 1–3% of responses.

We assessed the prevalence of COVID-19 vaccine acceptance intent by sociodemographic characteristics (Table 2). The survey indicates that 79% of the respondents reported they plan to accept a COVID-19 vaccine when one becomes available. With regard to the demographic characteristics associated with COVID-19 vaccination intention, intent to get vaccinated was quite similar between females and males; 80% of females intend to get a vaccine and 79% of males intend to get a vaccine. Across all age groups, a higher proportion of respondents in the older age groups were willing to get vaccinated than those in the younger age groups: 93% of respondents older than 60 years old expressed vaccine intent, compared to only 73% of respondents younger than 30 years old. Similarly, there was an increasing trend of vaccine acceptance with better perception of one’s own health. As for area of dwelling, there were small differences in vaccine acceptance with 80% of urban residents reporting intent to receive COVID-19 vaccine compared to only 76% of rural residents. Intent to vaccinate was higher among those who obtained less than secondary education (82%) than those with a secondary (73%) or tertiary education (79%).

**Table 2:** Demographic characteristics vaccination intention (n = 2,180).

Demographic variable	Likely to get vaccine^A^ (%)	Not likely to get vaccine (%)
Gender		
Female	79.7%	20.3%
Male	78.5%	21.5%
Other	0.00%	100%
Education		
Less than secondary	79.1%	20.9%
Secondary	72.9%	27.1%
Tertiary	82.4%	17.6%
Age		
Under 20	69.7%	30.3%
20–30 yr	73.8%	26.2%
31–40 yr	82.1%	17.9%
41–50 yr	89.7%	10.3%
51–60 yr	90.3%	9.7%
61–70 yr	91.8%	8.2%
71–80 yr	95.0%	5.0%
Over 80	100.0%	0.0%
Area of dwelling		
City	79.5%	20.5%
Town	78.8%	21.2%
Village or Rural	75.6%	24.4%
Own Health		
Poor	67.7%	32.3%
Fair	75.5%	24.5%
Good	79.5%	20.5%
Very Good	79.0%	21.0%
Excellent	80.0%	20.2%

Missing data not included. Missing data accounted for 1–3% of responses.

^A^ Responses to the vaccination intention question was dichotomized into “will get vaccinated” (n = 2058) versus “unsure/will not get vaccinated” (n = 556).

Examining the prevalence of COVID-19 vaccination intention by other characteristics, we found that while overall perception of COVID-19 community risk did not vary (13.5% low risk perception vs. 13.4% high risk perception), intent to get vaccinated differed. Out of 109 respondents who believed COVID-19 was not at all dangerous to their community, only 72% expressed vaccine intent. However, 86% of the 108 respondents who believed COVID-19 was extremely dangerous reported intent to receive COVID-19 vaccine.

**Fig 1:**
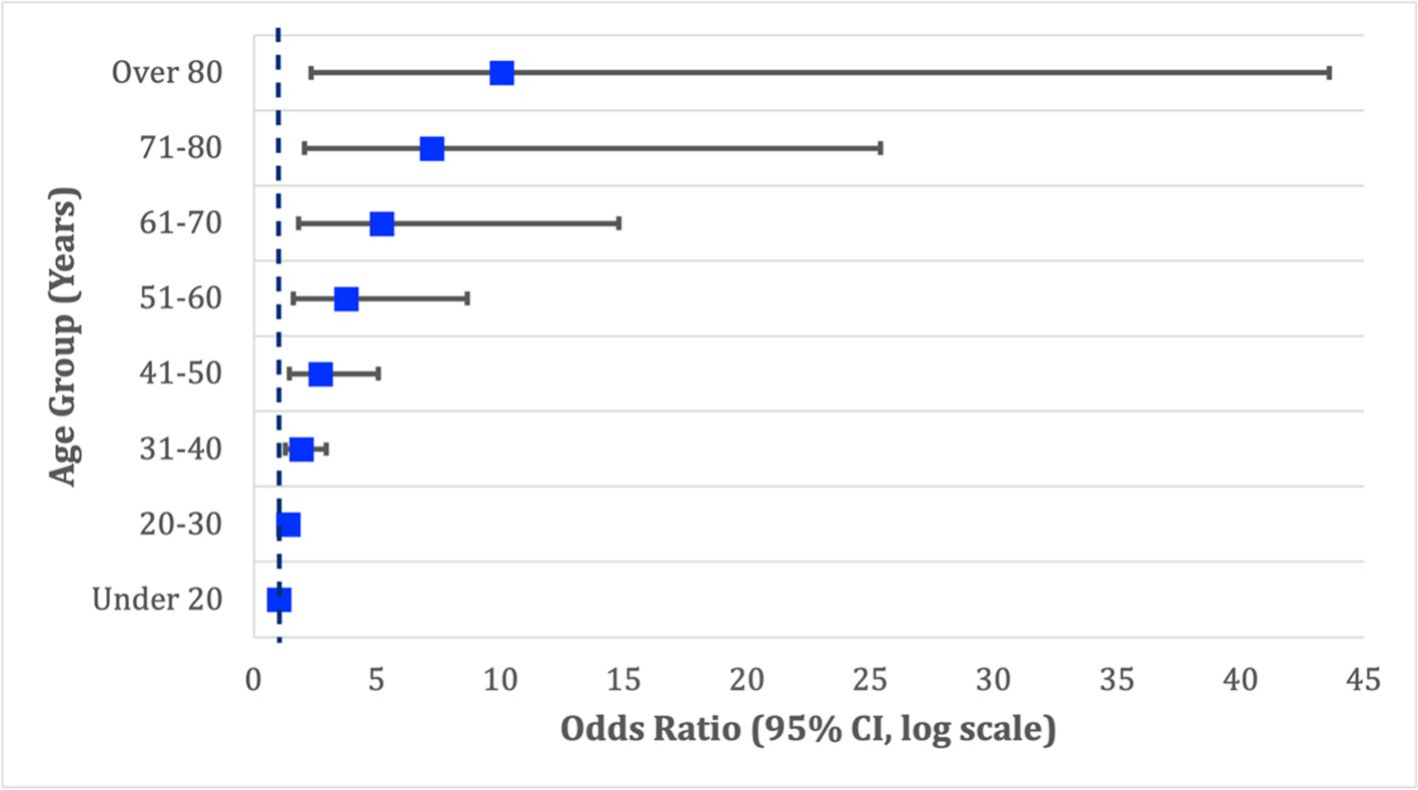
Forest plot of odds ratios for COVID-19 vaccination intention by age group.

Knowledge of existing treatments, perceived community risk, preventive measures taken (washing hands, social distancing, and none), and future behaviors (masks and social distancing) were significant predictors of COVID-19 vaccine intent only in the unadjusted binomial logistic regression model. Results from the full binomial logistic regression model predicting intention to vaccinate against COVID-19 are presented in Table 3. In the adjusted model, significant predictors of vaccination intention included age, perceived COVID-19 risk to community, and willingness to social distance in the future. With each 10-year increase in age group, the odds of intending to accept a COVID-19 vaccine increase by nearly 40% (OR [odds ratio] 1.39, 95% CI [95% confidence interval] 1.13–1.72) (Fig 1). Respondents more than 80 years old reported an odds of accepting a vaccine 10 times greater than those younger than 20 years old. Furthermore, respondents who believed the COVID-19 risk was extremely high in their communities had an odds of vaccine intention 1.24 times greater than those who believed COVID-19 was not dangerous at all to their communities (OR 1.24, CI 1.05–1.48). Similarly, respondents who reported they were likely to socially distance over the next two weeks showed increased odds of vaccine acceptance compared to those who were not as likely to socially distance (OR 1.22, CI 1.00–1.50).

**Table 3:** Crude and adjusted logistic regression analysis of independent variables of COVID-19 vaccination intention.

	Unadjusted OR (95% CI)	Adjusted OR* (95% CI)
Gender^A^		
Female	REF	REF
Male	0.90 (0.698–1.16)	1.08 (0.638–1.81)
Education		
Less than secondary	REF	REF
Secondary	0.92 (0.756–1.13)	1.11 (0.656–1.86)
Tertiary	0.85 (0.572–1.28)	1.22 (0.430–3.46)
**Age** *		
Under 20	REF	REF
20–30 yr	1.50 (1.36–1.65)	1.39 (1.13–1.72)
31–40 yr	2.25 (1.85–2.72)	1.93 (1.27–2.94)
41–50 yr	3.38 (2.52–4.49)	2.69 (1.43–5.04)
51–60 yr	5.08 (3.42–7.41)	3.73 (1.61–8.65)
61–70 yr	7.62 (4.65–12.23)	5.19 (1.82–14.8)
71–80 yr	11.44 (6.33–20.2)	7.21 (2.05–25.4)
Over 80	17.17 (8.61–33.3)	10.03 (2.31–43.6)
Area of dwelling		
City	REF	REF
Town	0.90 (0.798–1.02)	1.15 (0.870–1.51)
Village or Rural	0.81 (0.647–1.04)	1.32 (0.757–2.29)
Own Health		
Poor	REF	REF
Fair	1.09 (0.988–1.20)	1.13 (0.921–1.38)
Good	1.18 (0.976–1.44)	1.27 (0.848–1.91)
Very Good	1.29 (0.964–1.73)	1.44 (0.781–2.63)
Excellent	1.40 (0.953–2.07)	1.62 (0.720–3.64)
Know a positive case		
No	REF	REF
Yes	1.13 (0.934–1.36)	1.45 (0.938–2.23)
Prefer not to say	1.27 (0.872–1.85)	2.09 (0.880–4.97)
Knowledge of Existing Treatment		
There is currently not drug treatment or vaccine for COVID-19	REF	REF
There is a drug to treat to COVID-19	0.85 (0.760–0.956)	0.99 (0.765–1.29)
There is a vaccine for COVID-19	0.73 (0.578–0.914)	0.98 (0.585–1.65)
There is both a drug for treatment and a vaccine for COVID-19	0.62 (0.439–0.874)	0.98 (0.448–2.12)
I am unsure which is correct	0.53 (0.334–0.835)	0.97 (0.342–2.73)
**Community risk** *		
Not at all dangerous	REF	REF
Slightly dangerous	1.25 (1.08–1.43)	1.24 (1.05–1.48)
Moderately dangerous	1.55 (1.16–2.04)	1.55 (1.10–2.18)
Very dangerous	1.93 (1.26–2.92)	1.92 (1.15–3.22)
Extremely dangerous	2.40 (1.36–4.18)	2.39 (1.20–4.75)
Washing Hands		
No	REF	REF
Yes	1.31 (1.05–1.64)	0.70 (0.405–1.19)
Face Mask		
No	REF	REF
Yes	1.55 (1.20–2.01)	0.80 (0.377–1.72)
Social Distancing		
No	REF	REF
Yes	1.81 (1.46–2.28)	1.31 (0.812–2.10)
None of these		
No	REF	REF
Yes	0.44 (0.297–0.646)	0.39 (0.127–1.18)
Masks		
Never	REF	REF
Rarely	1.48 (1.33–1.64)	1.16 (0.912–1.47)
When convenient	2.19 (1.77–2.69)	1.34 (0.832–2.16)
Almost always	3.24 (2.35–4.41)	1.55 (0.759–3.17)
Always	4.78 (3.13–7.23)	1.79 (0.692–4.66)
**Future Distance** *		
Never	REF	REF
Rarely	1.40 (1.28–1.54)	1.22 (0.997–1.50)
When convenient	1.97 (1.64–2.37)	1.50 (0.994–2.25)
Almost always	2.76 (2.10–3.65)	1.83 (0.991–3.38)
Always	3.86 (2.68–5.62)	2.24 (0.988–5.06)

REF = reference category.

* **p < 0.05.**

A”Other” category of gender not included for data analysis because too few cases to allow for meaningful analyses.

## Discussion

This study revealed that 79% of respondents intend to get vaccinated; these results are congruent with the only other peer-reviewed study on vaccine acceptance conducted in Bangladesh [20]. The prevalence of vaccine acceptance from our study was similar to that identified in an India population-based study [31]. However, these results are higher than the actual vaccination rate in Bangladesh (25%), which may be due to inadequate vaccination capacity, dose shortage, and other supply-related issues [32, 33].

Our study indicates that age, risk perception, and willingness to social distance are significant positive predictors of vaccination intention among Bangladeshi adults. We found that intent to be vaccinated increased substantially with age—with older respondents more likely to intend to be vaccinated than younger respondents—and that concern about severity of COVID-19 in one’s community was associated with vaccination intent. While our results regarding age contradict findings from other studies, conclusions about age were not statistically significant by the other vaccine intention study conducted in Bangladesh [20]. Furthermore, our sample had a small representation of older adults, which suggests that more evidence is needed for a stronger conclusion. However, the implications of this finding are noteworthy, as 6% of households in Bangladesh have members that are 65 years of age or older [34], meaning the vaccine behaviors of older adults will impact millions of families.

This is one of the first studies in Bangladesh that examined the prevalence of vaccine acceptance and its association with sociodemographic factors. It is clear there is a need and opportunity for targeted outreach and messaging. As has been seen in other countries, recent surges have impacted younger age cohorts, especially as vaccination is prioritized for older populations at greatest risk of severe illness and death. Although vaccination intent is generally high, closing gaps among those who express hesitancy will be critical especially in a country like Bangladesh where there is limited health infrastructure, high rates of extreme poverty, and high population density, all factors that increase COVID risk exposure. To keep community transmission low, it is imperative that the country develop and implement targeted risk communication efforts for younger adults to increase awareness of the severity of the disease and confidence in available vaccines. Given Bangladesh’s common extended family structure, the difference in intent between younger and older adults will be important in developing messaging around protecting the elders and increasing vaccine acceptance among younger adults, particularly because younger adults and older adults are in frequent contact with one another [34].

### Limitations

This work is not without limitations. Since our survey was conducted after Bangladesh began mass COVID-19 vaccination campaigns in February, there may have been significant changes in perceived risk of disease and perceived safety of COVID-19 vaccines from before campaigns started. There is potential for biased data, as online surveys may not be able to reach certain types of participants, particularly those that do not have Internet access or do not feel comfortable navigating online surveys. Self-selection bias and self-report bias are also likely. As the data were collected by outside partners, we had no control over the variables of interest, sampling frame, and recruitment methods. Religion plays an important role among South Asian people, but we were not able to explore the connection between religion and vaccination intent since the original data did not ask questions pertaining to religion. Also, women may have been under-represented in the survey. However, these limitations were partially addressed via the sampling and weighting methods used.

## Conclusions

As countries in the lower-income context are slowly starting to receive COVID-19 vaccine doses, this is a critical time to enhance country readiness for COVID-19 vaccine delivery through targeted communication and policy actions. Countries must be able to create demand for COVID-19 vaccines, given that they are new vaccines and transmission is ever-changing. This will be particularly challenging as there is low trust in health care systems, and people have been living in uncertainty for an extended period. As such, understanding attitudes toward COVID-19 vaccines, and then using this information to subsequently develop tailored approaches for communication strategies will be paramount for successful vaccine uptake.
